# Management of Blunt Pancreatic Trauma in Children: A Persistent Controversy—Case Report and Comprehensive Literature Review

**DOI:** 10.3390/children11010135

**Published:** 2024-01-22

**Authors:** Igor Sukhotnik, Neta Cohen

**Affiliations:** 1 Department of Pediatric Surgery, Dana Dwek Children’s Hospital, Tel Aviv Sourasky Medical Center, Affiliated to the Faculty of Medicine, Tel-Aviv University, Tel Aviv 69978, Israel; 2 Pediatric Emergency Department, Dana Dwek Children’s Hospital, Tel Aviv Sourasky Medical Center, Affiliated to the Faculty of Medicine, Tel-Aviv University, Tel-Aviv 64239, Israel; netaco@tlvmc.gov.il

**Keywords:** pancreatic trauma, children, non-operative management, diagnosis, main duct injury, pancreatectomy, pseudocyst

## Abstract

Blunt pancreatic injury (BPI) is relatively uncommon in children, and is associated with relatively high morbidity and mortality, especially if diagnosis is delayed. The aim of this report is to review the literature regarding controversial questions in the early diagnosis and management of pediatric BPI. A representative case of blunt pancreatic trauma in a six-year-old girl with delayed diagnosis and intraoperative and postoperative complications was described. A systematic search of databases and the grey literature in Scopus and Web of Science using relevant keywords was conducted. A total of 26 relevant articles published in last 5 years were found in PubMed. Although early CT performance is considered part of initial pancreatic trauma workup, the sensitivity of CT for detecting main pancreatic duct injuries in children is relatively low. MRCP and ERCP (if available) are useful for assessing ductal injury and should be performed when the status of the pancreatic duct is unclear on the CT. Most patients with low-grade pancreatic damage may be treated conservatively. Although surgery involving distal pancreatectomy remains the preferred approach for most children with high-grade pancreatic injury, there is growing evidence to suggest that non-operative management (NOM) is safe and effective. Most pancreatic pseudo cysts following NOM had relatively mild complications, and most resolved spontaneously. For those children who do require surgery, a conservative operative approach with the least risk is advocated. In conclusion, the optimal management for pediatric pancreatic trauma is controversial. Further clinical trials are required to generate clinical practice guidelines on pancreatic trauma in a child population.

## 1. Introduction

Non-operative management (NOM) of isolated solid organ (ISO) injury (spleen, liver, pancreas and kidney) in hemodynamically stable children has been generally successful, and is considered as a standard practice [1]. However, there is considerable variation in the therapeutic management algorithms used by individual pediatric surgeons. While isolated splenic and hepatic trauma can be managed conservatively in 90–95% of pediatric patients [2], children who sustained blunt pancreatic injuries (BPIs) were reportedly more likely to fail NOM compared with those who underwent other ISO traumas [3]. BPIs are relatively rare, occurring in 3% to 12% of children who sustained blunt abdominal trauma, and they are associated with high rates of morbidity and mortality. The most common injury mechanisms in this population is bicycle handlebars and dirt bikes (50%) [4]. Other mechanisms include abdominal trauma during sport activities; car accidents; fall from height; and collision during play [4]. The high morbidity rate of pancreatic trauma is directly attributed to delayed diagnosis, incorrect classification of the injury, or delays in treatment. While adults with BPI are most commonly treated surgically, there is still no consensus with respect to the optimal management of pancreatic trauma in pediatric patients. Some trauma centers favor and document the efficacy and safety of NOM for virtually all pancreatic injuries, including duct disruption, while others advocate aggressive operative pancreatic management with debridement or a distal pancreatectomy [1,2,3].

We describe a case of a pancreatic injury in a child whose diagnosis of pancreatic trauma was delayed and whose surgery bore several complications. We also present a literature review to address some controversial issues that arose during the management of this patient.

## 2. Materials and Methods

### 2.1. Case Report

**Figure 1:**
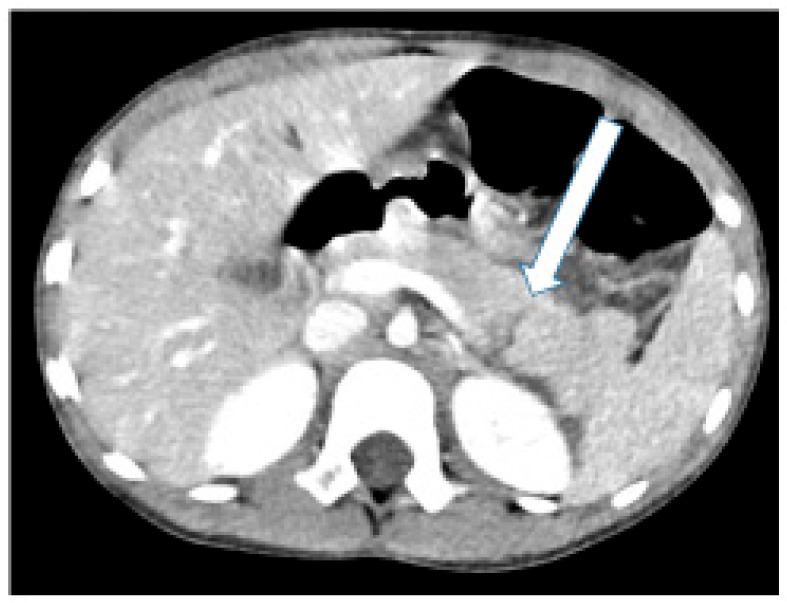
Abdominal computed tomography scan demonstrating a pancreatic injury to the left of the superior mesenteric vein (arrow).

A 6-year-old girl arrived at the Pediatric Emergency Department 24 h after she had fallen on her bicycle handlebar and experienced discomfort in her left upper abdomen. Her medical history revealed no prior conditions or medications. The PED assessment by the medical team followed the advanced trauma life support (ATLS) protocol. Her airway was clear, and breathing was normal. She had tachycardia (heart rate: 140 bpm), but good capillary refill and normal perfusion. Blood pressure was normal for her age. She rated her pain at 4/10 on a visual analogue scale. There was a reddish bruise between her lower left chest and upper left abdomen, accompanied by local mild tenderness. She displayed no neurological deficits. The focused assessment with sonography in trauma (FAST) examination revealed a small amount of fluid in the suprapubic area. A contrast-enhanced computerized tomographic (CT) scan revealed a laceration in the distal pancreas, with limited visibility of the main pancreatic duct (MPD) (Figure 1).

Blood tests indicated high levels of amylase and lipase (815 and 808 U/L, respectively), while liver enzyme levels were normal. Her complete blood count levels, including hemoglobin, white blood cell count, and platelet count, fell within normal ranges. She was admitted to the pediatric surgical floor for conservative treatment consisting of fasting (NPO) and parenteral fluid therapy. Magnetic resonance cholangiopancreatography (MRCP) would be considered based upon her clinical progress.

Two days after admission and following an oral nutrition trial, the patient developed fever (38.3 °C), vomiting, and increased inflammatory markers. An abdominal ultrasound revealed a structured 3.7 × 4.5 × 5.1 cm collection with multiple septations in the distal pancreas. Pancreatic injury was defined as grade III (MPD injury to the left of the superior mesenteric vein), and she was taken to the operating room for a laparoscopic distal pancreatectomy. Upon opening the lesser sac, a collection containing opaque contents was observed near a deep laceration between the body and tail of the pancreas. The pancreas appeared swollen, thickened, and inflamed at the spleen’s portal, with the adjacent stomach wall showing signs of edema and the nearby retroperitoneum showing infiltration. At this point, the surgical team decided that spleen-sparing surgery was not feasible, and the surgical approach involved exploring the lesser sac to identify the pancreatic laceration. Dissection along the greater curvature of the stomach was performed in order to detach the omentum and gastrosplenic ligament. The splenocolic, splenophrenic, and splenorenal ligaments were also dissected to separate them from the body of the spleen. The margins for pancreatectomy were set at 2 cm to the right of the area of laceration. The pancreas was separated at its lower, upper, and retroperitoneal boundaries along the detachment of the splenic artery and vein. Retroperitoneal separation of the pancreas and spleen was completed, and both were removed through an expanded umbilical trocar. A percutaneous catheter drainage (JP 10) was left at the surgical site for drainage of pancreatic collection.

The patient was transferred to the pediatric intensive care unit for one day, with management consisting of continuous monitoring of percutaneous pancreatic collection drainage and nasogastric tube and urinary output. Subcutaneous somatostatin was administered to address a low-output pancreatic fistula. Parenteral fluids, proton pump inhibitors (PPI), and antibiotic treatment were also provided. On the second postoperative day, the patient began oral nutrition and showed favorable absorption. She was then transferred to the surgical floor, where gradual improvement in her condition was observed, leading to a transition to complete oral nutrition.

At 16 days since the time of injury and 14 days following the surgery, the patient was discharged home while retaining an open percutaneous pancreatic fistula external drain. She was also prescribed an oral PPI, received preventive antibiotic treatment, and was scheduled for vaccination (as routine prevention for patients following splenectomy). Twenty-five days post-discharge, she was admitted to the surgical floor for removal of the percutaneous pancreatic fistula drain. The procedure was completed without any complications and was well-tolerated by the patient. The final outcome was entirely favorable and free of recurrent fistula or pseudocyst formation throughout the 12-month follow-up period. This case is reported in accordance with the CARE guidelines [5]. All of the patient’s information has been anonymized.

### 2.2. Literature Review

This report identified 3 main problems and controversies concerning the management of pediatric BPIs: (1) What are the best management guidelines for preventing delayed diagnosis following admission among children with isolated BPI; (2) What is the role of NOM of BPI with MPD involvement in children, and what are the outcomes and costs associated with NOM versus operative treatment of high-grade pediatric BPIs; (3) Is distal pancreatectomy superior to drainage procedures in Grade III BPIs in children? In order to answer these questions, an advanced search in PubMed including the keywords “blunt pancreatic trauma”, “children”, “non-operative management”, “early diagnosis”, “distal pancreatectomy”, and “complications” was performed. We provide a brief summary of the important aspects and options for BPI in children which may be considered at the time of evaluation and in management decision-making.

## 3. Results and Discussion

### 3.1. Blunt Pancreatic Trauma in Children—General

Blunt trauma represents the primary cause of pancreatic injury in the pediatric population. Pancreatic trauma in children remains a major challenge for emergency physicians as well as general and pediatric surgeons. Its rate of occurrence is 0.2–2%, and it contributes to 0.3% of all childhood injuries [1,6,7]. The mortality rate associated with BPI remains low, ranging between 4.7–5.3%, with most fatalities linked to concurrent injuries. While there are established protocols for diagnosing and surgically managing pancreatic injuries in adults, the approaches to handling high-grade BPI involving the major pancreatic duct in children remain a subject of debate. In 2022, The Western Trauma Association (WTA) published clinical practice guidelines on pancreatic trauma in the adult population [8]. The WTA evaluation and management algorithm applies to the diagnosis and management of adult patients with BPI. Since delayed diagnoses can result in increased morbidity and mortality of up to 62% of patients [8], the WTA Committee recommends early performance of CT as part of the initial trauma workup. Imaging findings of transection of the pancreas, disruption of the MPD, or of a large amount of peripancreatic fluid mandate operative exploration. When imaging findings are not sufficiently clear-cut, other investigations may be useful, and they include serial abdominal examinations, serum amylase and lipase enzyme levels, MRCP, endoscopic retrograde cholangiopancreatography (ERCP), and transduodenal pancreatography. The major determinant in management decisions in adults with BPI is the presence or absence of injury to the main pancreatic duct (MPD). Since low-grade pancreatic trauma (Grades I and II) are contusions and lacerations that spare the pancreatic duct, they are mostly managed conservatively. In adult patients with low-grade injuries who have indications for laparotomy, drain placement to control the leakage is recommended only if there is pancreatic capsule disruption. In accord with the WTA algorithm, most adults with “high-grade” pancreatic injuries (Grades III = MPD injury to the left of the superior mesenteric vein [SMV], Grade IV = MPD injuries to the right of the SMV, and Grade V = involving disruption of the head of the pancreas) require definitive surgical treatment to avoid duct-related complications that carry a morbidity of up to 60% [8].

There are no clear-cut guidelines for the initial management of BPI in children among whom the diagnosis, classification, and treatment remains a challenge. Non-operative management of ISO injuries in stable children is also pertinent to the management of BPI. The BPI we report in a six-year-old girl, which manifested with unclear clinical presentations of an MPD injury, resulted in delayed diagnosis and surgical intervention. During her operation, spleen-sparing surgery was not feasible, and she underwent a distal pancreatectomy and splenectomy. Postoperatively, she developed a pancreatic fistula that was treated by external catheter drainage and required total parenteral nutrition (TPN) for two weeks, and repeat administration of Sandostatin. The percutaneous pancreatic fistula drain was removed one month later, and the fistula closed spontaneously. The child’s outcome was ultimately favorable, with no recurrence of symptoms during the 12-month follow-up period.

### 3.2. Early Diagnosis—Pitfalls

Early diagnosis of pancreatic trauma is key to optimal management, but it remains a challenge even with more advanced imaging modalities. Traumatic BPIs are associated with high morbidity and mortality rates in both adults and children, making it crucial to minimize time for diagnosis and appropriate intervention. Due to its protected retroperitoneal location, injuries of the pancreas are uncommon in children and are often misinterpreted. The symptoms and physical signs of BPI in children may be nonspecific or even absent, and are frequently overlooked for not being readily apparent on initial examination. Additionally, abdominal symptoms such as abdominal pain, nausea, and vomiting do not always correlate with trauma severity.

Table 1 summarizes a current (the past 5 years) literature review of publications on the early diagnostic tools during initial management in children with BPI.

US is commonly used to detect intra-abdominal organ injury. It is commonly available in emergency rooms, and the imaging study is routinely part of the initial assessment of children with blunt abdominal trauma. US may serve as a good rapid screening procedure, particularly in patients too unstable to undergo an abdominal CT scan. However, US is limited by its low sensitivity and specificity when determining acute pancreatic injuries. The reported sensitivities for the detection of pancreatic injuries by US ranged from 27% to 96% [15]. Zhang et al. reported a 68% accuracy rate for detecting pancreatic injury in 51 children by early US (Table 1) [9]. Ultrasound imaging, however, cannot provide valuable information regarding the size, location, and characteristics of BPIs [14].

The WTA Committee recommends early CT as part of an initial pancreatic trauma workup in adults [8]. CT scanning has been the diagnostic imaging method of choice to detect BPI in adult and children for more than three decades. It is highly accurate in diagnosing pancreatic damage, thereby lowering the rate of missed or delayed diagnoses of BPI, leading to decreased morbidity and mortality, and serving as an important factor in determining the need for surgical treatment. However, several studies have reported low sensitivity of CT ranging from 38% to 61% for diagnosing MPD injuries in children [1,2,3,4,5,6,7,8,9,14,15,16] (Table 1). Recent advancements in technology (multidetector CT technology [MDCT]) have enabled improved detection of these injuries. Phelan et al. reviewed the findings of 16-MDCT and 64-MDCT studies from 22 centers (206 pediatric patients with BPI) and compared them to the operative findings. Those authors reported that the sensitivity for MPD injury was 54% for 16-MDCT and 52% for 64-MDCT, although the specificity was higher (95% and 90%, respectively) [16]. In addition, they observed that the overuse of CT in blunt abdominal trauma in children leads to inefficient care and radiation-induced malignancies. Therefore, to maximize precision and minimize the overuse of CT, several professional societies and organizations proposed clinical prediction rules for determining the use of radiographic imaging after traumatic injuries [17].

MRCP is a useful alternative diagnostic modality thanks to it lacking the need for ionizing radiation, its panoramicity, its affording the possibility to avoid the use of contrast media, and its ability to properly evaluate even small pancreatic ductal disruptions. MRCP is also highly sensitive in distinguishing between different types of BPI. Rosenfeld et al. recently compared the accuracy of CT and MRCP for identification of MPD disruption in BPI in children (Table 1) [14]. Data were obtained from eleven pediatric trauma centers. The results of this study showed that MPD visualization and duct disruption were visualized more often on MRCP than on CT, but the overall MRCP score (duct visibility, duct disruption, pancreatic parenchymal injury, and secondary findings [e.g., peri-pancreatic fluid collections and free intraperitoneal fluid]) for determining duct integrity was not better than that of CT (38% vs. 62%, respectively, *p* = NS). In a large series by Zhang et al. (51 children with BPI), 46 patients (90.2%) underwent abdominal CT, 31 patients (60.8%) underwent enhanced CT, and 12 patients (23.5%) underwent MRCP. Those authors reported a 77% accuracy rate for CT and a 100% accuracy rate for MRCP in identifying MPD damage [9]. Ibrahim et al. recently presented their experience with CT and MRI in pediatric pancreatic trauma and correlated the imaging grade of pancreatic injury with management and outcome. Those authors reported a 93% accuracy for CT (27 patients) and a 100% accuracy for MRCP (10 patients) [13].

During the last decade, contrast-enhanced ultrasound (CEUS) has been considered an appealing alternative to contrast-enhanced CT in the evaluation of children with blunt abdominal trauma, mainly with respect to the potential reduction in the use of ionizing radiation and contrast media [18]. Pancreatic lacerations and fractures appear on CEUS as non-enhancing or hypoenhancing defects in both the arterial and venous phases of enhancement, and are frequently seen to involve the pancreatic capsule [11]. However, CEUS has some limitations, particularly in the assessment of small pancreatic lesions, in the evaluation of mild ductal disruptions, and in the detection of vascular complications. In a recent comparative study, Miele et al. compared the usefulness and the feasibility of MRI and CEUS in the follow-up of patients with low-grade blunt abdominal trauma. Those authors showed that MRI enabled a better assessment of injuries than CEUS while also allowing the determination of the temporal stage of the lesions [19].

The specificity of ERCP in the identification of MPD disruption is very high in most cases, and it has the added benefit of guiding therapeutic intervention. However, the invasive nature of ERCP and the lack of widespread availability for the pediatric population at many institutions limit its utility. Additionally, ERCP does not allow for evaluation of the pancreatic parenchyma and surrounding tissue damage, nor can it detect pancreatic duct disruption distal to an obstruction. In a recent trial, Gong et al. [10] discussed the usefulness and safety of ERCP in traumatic pancreatic injury in children, 48% of whom had BPI and underwent ERCP. Those authors concluded that ERCP was indicated for both diagnostic purposes since imaging findings on CT and US are not as clear-cut as therapeutic purposes after pancreatic duct injury had been identified on radiologic imaging. ERCP was performed for therapeutic purposes in four of their patients. Those authors showed that the diagnostic accuracy of radiologic injury grade (correlating to the final injury grade) was about 61%, while ERCP had a diagnostic accuracy rate of 86%. They concluded that ERCP can be usefully and safely performed in children with BPI for both diagnostic and therapeutic purposes.

### 3.3. Treatment Strategy—Non-Operative versus Operative Management

The two main strategies for managing pediatric pancreatic trauma are non-operative and operative. The non-operative method typically involves vigilant monitoring of the child’s clinical status, frequent imaging through modalities (CT, ultrasound, MRCP), continuous assessment of amylase and lipase levels, nasojejunal feeds, total parenteral nutrition, octreotide, drainage (radiological and endoscopic), and ERCP [8]. Table 2 presents a summary of publications related to the management of pancreatic injury in children that appeared during the past 5 years and that addressed two other controversial questions: (1) operative versus non-operative treatment of children with BPI, and (2) aggressive (pancreatectomy with or without splenectomy) versus more conservative surgical management in children for whom operative treatment is indicated.

Since the first description of conservative treatment of children with splenic injury in the early 1950s at the Hospital for Sick Children in Toronto, the same concept has been successfully applied to most blunt injuries of the spleen, liver, kidney, and pancreas in children nearly seven decades later [1]. Our colleagues in adult trauma care have slowly acknowledged this success, and are applying many of the principles learned in pediatric trauma to their patients. However, there are several areas of disagreement regarding the management of pancreatic trauma between general and pediatric surgeons. Those disputes are related mainly to the more conservative philosophy in the management of children who sustained blunt abdominal trauma. There is no argument that the anatomic, immunologic, and physiologic differences between pediatric and adult trauma patients must be taken into consideration and must be incorporated into treatment protocols. There is also consensus regarding non-operative treatment protocols for low-grade pancreatic trauma (Grades 1 and 2) in both adult and pediatric populations [1,8]. The main disagreements concern the management of high-grade pancreatic trauma (Grade 3–5). Discussions regarding operative versus non-operative management of children with pancreatic trauma have a long history. More than 20 years ago, two trauma centers (Toronto and San Diego) reported their experience with different methods and different management protocols of managing blunt traumatic pancreatic injuries in children. Canty and Weinman (San Diego) reported 18 patients with major ductal injuries over a 14-year period. Distal pancreatectomy was carried out in eight patients (44%) with distal duct injuries, while nonoperative management was followed in patients with proximal duct injuries. Two of these patients underwent successful ERCP with duct stenting, and seven of them either developed pseudo cyst formation that spontaneously resolved (2 patients) or were treated through delayed cystogastrostomy (5 patients) [21]. Around the same time, the experience summarized in three reports from the Toronto trauma center was markedly different. Shilyansky et al. reported 35 consecutive children with pancreatic injuries treated over a period of 10 years [22]. Twenty-eight children (80%) who sustained BPI were treated non-operatively, and 14 of them had ductal transection or a pancreatic pseudo cyst. Ten other children developed pseudo cysts that were successfully managed non-operatively, although percutaneous aspiration or drainage was required in 6 of them. Those authors concluded that NOM is effective and safe for virtually all pancreatic injuries, including those with duct disruption.

Now, more than two decades later, the controversy still exists. Several reports from different major pediatric trauma centers are in clear opposition. Some favor and document the efficacy and safety of non-operative management for virtually all BPIs, including MPD injury. Others advocate aggressive surgical management with debridement, drainage, or pancreatectomy. Advocates of surgical intervention for BPIs cite their concern about missing associated abdominal injuries if no operation is performed. Kopljar recently published a systematic review and meta-analysis of initially non-operative versus initially operative treatment in children with BPI (Table 2) [20]. The 42 studies in this review included 1754 patients, of whom 1095 were initially managed non-operatively and 659 were managed operatively. While non-operative treatment was successful in 87% of patients, that group presented with a significantly higher percentage of pseudocyst formation (24.9% vs. 7.4% for the operated group), a significantly lower percent of pancreatic fistula formation (1.0% vs. 7.6%), slightly shorter (non-significantly) length of stay, slightly longer (non-significantly) duration of TPN, and unchanged risk of re-admissions. Data meta-analysis for patients with grade III or higher pancreatic injury revealed a significantly higher risk of pseudocyst formation in the NOM group (95% vs. 33.8% for the non-NOM group) and unchanged risk of pancreatic fistula formation. There was no significant difference in the risk of mortality between groups.

In another clinical trial, 36 children admitted with pancreatic trauma were analyzed for their presentation, management, and outcome [23]. They all presented with sequelae of ductal disruption with or without a pseudocyst, ascites, or pleural effusion. Thirty-four patients were treated non-operatively by means of nasojejunal feeds, TPN, octreotide, drainage (radiological and endoscopic), and ERCP (12%). Only two patients underwent surgery consisting of cystojejunostomy and peritoneal lavage in one each. Altogether, 59.3% of patients fully recovered and 40.6% developed pancreatic pseudocysts that were treated successfully by either external or internal drainage. Those authors concluded that multi-disciplinary NOM was effective for managing high-grade pancreatic injury in 94% of their study children, with 75% of them requiring radiological or endoscopic interventions. In addition, 40% of their patients later developed structural changes, but only one-half were symptomatic. Zhang et al. recently reported 51 children with BPI [9], among whom only two underwent surgical procedures, both with drainage with/without debridement. However, 47% of their patients developed pancreatic pseudocysts (75% with high-grade injuries [HGI] and 42% with low-grade injuries [LGI]). All of their patients were successfully treated by ultrasound-guided external drainage or received laparoscopic external drainage with good long-term clinical outcomes. Gong et al. recently demonstrated that only 43% of their study children with Grades 3 and 4 pancreatic injuries were treated operatively, with relatively low rates of late complications (19%) [10] (Table 2). Goldberg-Murow et al. [12] described the results of treatment among 11 children with HGI (Grade 3–5 patients and Grade 4–6 patients). Five of those children were treated conservatively. All of their NOM patients developed a pancreatic pseudocyst while only one-third of the operated children developed pseudocyst formation. However, most of those pseudocysts had relatively benign complications, and most resolved spontaneously with no need for surgical intervention. Two children underwent percutaneous drainage due to the large size of the cysts. Hospitalization time was similar for the operative and non-operative groups. Ibrahim et al. [13] described 28 pediatric patients with BPI, two of whom did not survive. Sixteen of the remaining patients had LGI and were treated conservatively. Ten other patients had HGI, and seven of them who had initially been treated conservatively underwent ERCP with or without stent placement. Five patients developed a pancreatic pseudocyst and were treated by external or internal drainage.

### 3.4. Operative Treatment Objectives

Pancreatic injury severity is classified by the American Association for the Surgery of Trauma (AAST) scale. Injuries with AAST grades Ⅰ and Ⅱ are categorized as being LGIs, whereas those meeting AAST grades Ⅲ to Ⅴ fall into the HGI category [24]. The WTA Committee defines Grade III of pancreatic injury as an MPD injury to the left of the superior mesenteric vein, and it recommend distal pancreatectomy as the gold standard in adults with this kind of damage. Although most reports in children have shown a relatively low frequency of Grade 3 BPI (10% according to Zang et al. [9], and 22.6% according to Gong et al. [21]), a recent study by Cattelani et al. reported that 17 out of 20 children and 5 out of 10 adults had Grade III pancreatic trauma [4]. As mentioned earlier, children with Grade III pancreatic trauma can be treated non-surgically. In trauma centers that prefer operative management for most patients with Grade III, an additional controversial question is the preferred surgical approach: distal pancreatectomy or a more conservative procedure. According to the WTA algorithm for adults with BPI [8], pancreatic resection is recommended for patients with Grade IV injury when the required surgical expertise is available, and drainage is recommended when it is not available. Pancreaticoduodenectomy will generally be required for stable patients with Grade V injuries. For unstable patients, or if adequate surgical expertise is not available, damage-control surgery, drain placement, and ultimately a staged procedure, are recommended. There are no similarly clear recommendations for the pediatric population. However, extrapolating these data to children with pancreatic trauma and adhering to a more conservative philosophy for pediatric abdominal blunt trauma, a more conservative surgical management (damage control surgery, drain placement, staged procedure) may be safely followed for children with high-grade pancreatic injuries. Catellani et al. recently described their experience in the management of blunt pancreatic trauma in 30 patients with pancreatic trauma (10 adults and 20 children) that consisted of a laparoscopic approach [4]. The mean blood loss during the children’s operations was 75 mL, the mean hospital stay was 9 days, and the complication rate was 40%. Those authors concluded that laparoscopic management of BPIs in a hemodynamically stable child is feasible and safe when performed by an experienced laparoscopic pancreatic surgical team.

The efficacy and safety of stent placement during ERCP has been recently reported in children with BPI and MPD damage as an alternative to a surgical approach. Ishikawa et al. recently reported the efficacy of stent placement via ERCP in six children with pancreatic trauma. Stent placement was performed at a site proximal to the injury in four patients and across the injury in two patients. A pseudocyst or pancreatic fluid collection was detected in five patients. In the four patients with pancreatic duct injuries, only one of whom where the stent was placed across the injury was able to avoid surgery [25]. Those authors concluded that therapeutic ERCP with stent placement might be effective even if a patient has a pancreatic duct disruption, and therefore, early ERCP should be considered as a treatment option.

## 4. Conclusions

The optimal management for pediatric pancreatic trauma is controversial. A strong index of suspicion, adequate diagnostic tests, and a multidisciplinary approach are essential for early detection and appropriate treatment. Although early CT performance is considered part of the initial pancreatic trauma workup, the sensitivity of CT for detecting main pancreatic duct injuries in children is relatively low. MRCP and ERCP (if available) are useful for assessing ductal injury, and should be performed when the status of the pancreatic duct is unclear on the CT. Most patients with low-grade pancreatic damage may be treated conservatively. Although surgery involving distal pancreatectomy remains the preferred approach for most children with high-grade pancreatic trauma, there is growing evidence to suggest that non-operative management is safe and effective for managing high-grade pancreatic injury. Although most NOM children would develop a pancreatic pseudocyst, most of those pseudocysts have relatively benign complications, and most resolve spontaneously with no need for surgical intervention. For those children who do require surgery, a conservative surgical approach with the least risk is advocated.

**Figure 2:**
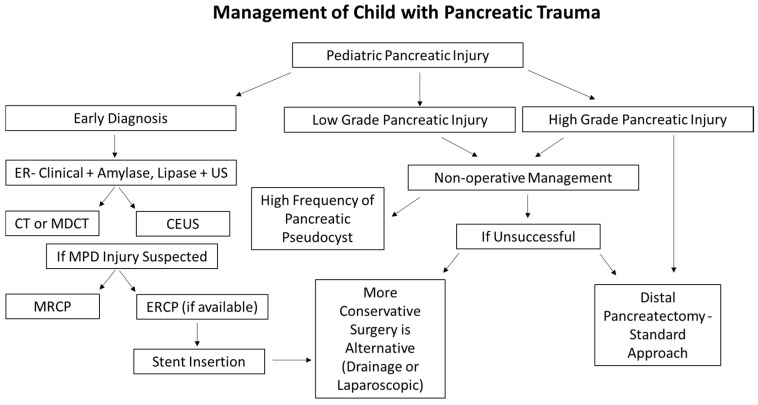
Protocol in the management of blunt pancreatic injury in children.

Figure 2 summarizes guidelines for clinical decision-making (diagnosis and investigation protocol, treatment options) in children with a blunt pancreatic injury.

## Figures and Tables

**Table 1:** The summary of previous literature regarding early diagnostics of pancreatic injury in children.

Authors/ Year	Number Patients (Mean Age/y)	Serum Amylase (No Pts/PV%)	Serum Lipase (No Pts/PV%)	US (No Pts/PV%)	CT (No Pts/PV%)	MRI (No Pts/PV%)	ERCP (No Pts/PV%)
Zhang et al. (2023) [9]	51 (7.3)	LGI—81% HGI—100%	LGI—53% HGI—100%	50 68%	45 77%	11 100%	0
Catellani et al. (2023) [4]	10 ad (28.2) 20 chld (10.5)	10 49%	N/A	N/A	10 90%	N/A 100%	N/A 100%
Gong et al. (2023) [10]	31 (11.7)	N/A	N/A	16 N/A	29 61%	N/A	15 86%
Everson et al. (2023) [11]	19 (13)	N/A	19 74%	1 N/A	19 79%	3 N/A	0
Goldberg-Murow et al. (2021) [12]	11 (9)	11 60%	N/A	FAST 11/ NA	11 90%	1 100%	0
Ibrahim et al. (2021) [13]	28 (7.14)	N/A	N/A	N/A	27/ 93%	10 100%	0
Rosenfeld et al. (2018) [14]	21 (7.8)	N/A	N/A	N/A	21 38%	NA 62%	0
Wiik-Larsen et al. (2020) [7]	10 (8.3)	9 67%	N/A	N/A	9 67%	3 100%	0

Abbreviations: Pts—patients, LGI—low grade injury, HGI—high grade injury, Pts—patients, PV—predictive values, N/A—not applicable; FAST—focused assessment with sonography in trauma; CT—Computed tomography, US—ultrasonography, MRI—Magnetic resonance imaging, ERCP—endoscopic retrograde cholangiopancreatography.

**Table 2:** The summary of previous literature (last 5 years) regarding management of pancreatic injury in children.

Authors/ Year	Number Patients (Mean Age/y)	NOM (% pts)	SPDP (%OM pts)	Drainage ± Suture (%OM pts)	Other (%OM pts)	Main Outcomes
Zhang et al. (2023) [9]	51 (7.3)	96%	0	4%	0	PP—75%HGI vs. 42%LGI
Catellani et al. (2023) [4]	20 pts (10.5)	Gr 2—100%	Gr 3—100%	0	Gr 4—DPS	PC—10% PF—10%
Gong et al. (2023) [10]	31 (11.7)	68% Gr 1,2—76% Gr 3,4—54%	N/A	N/A	N/A	EC—48% LC—19%
Everson et al. (2023) [11]	19 (13)	42% Gr 1,2—75% Gr 3,4—14%	22% All Gr 3	11% Gr 4	67%	PP—21% PF—5% Other 16%
Goldberg-Murow et al. (2021) [12]	11 (9) Gr 3—5 pts Gr 4—6 pts	45%	50%	33%	17%	PP—100% of NOM pts and 33% OM PF—11%
Ibrahim et al. (2021) [13]	28 (7.14)	70% HGI 100% LGI pts	2	0	2 (PP drainage)	PP—50% HGI pts
Wiik-Larsen et al. (2020) [7]	10 (8.3)	70%	33%	33%	33% DPS	N/A
Kopljar et al. Review+ meta-analysis (2021) [20]	42 studies 1754 pts	62%	N/A	N/A	N/A	87% success in NOM PP—24.9% vs. 7.4% PF—1% vs. 7.6% in NOM vs. OM

Abbreviations: NOM—non-operative management; OM—operative management; EC—Early complications; LC—Late complications; SPDP—spleen-preserving distal pancreatectomy; DPS—distal pancreatic-splenectomy; Gr 2,3,4—grade injury 2,3,4; PC—pancreatic collection; PF—pancreatic fistula, PP—pancreatic pseudocyst, LGI—low grade injury, HGI—high grade injury; pts—patients; N/A—not applicable.

## Data Availability

Not applicable.

## Abbreviations

NOM—non-operative management; ISO—isolated solid organ; BPI—blunt pancreatic injury; CT—computerized tomography; MRCP—Magnetic resonance cholangiopancreatography; US—ultrasonography; MPD—main pancreatic duct; HGPI—high grade pancreatic injury; MPD—main pancreatic duct; TPN—total parenteral nutrition; MDCT—multidetector CT; CEUS—contrast-enhanced ultrasound; ERCP—Endoscopic retrograde cholangiopancreatography; SPDP—spleen-preserving distal pancreatectomy; DPS—distal pancreatic-splenectomy; PC—pancreatic collection; PF—pancreatic fistula, PP—pancreatic pseudocyst.
